# Creating polar antivortex in PbTiO_3_/SrTiO_3_ superlattice

**DOI:** 10.1038/s41467-021-22356-0

**Published:** 2021-04-06

**Authors:** Adeel Y. Abid, Yuanwei Sun, Xu Hou, Congbing Tan, Xiangli Zhong, Ruixue Zhu, Haoyun Chen, Ke Qu, Yuehui Li, Mei Wu, Jingmin Zhang, Jinbin Wang, Kaihui Liu, Xuedong Bai, Dapeng Yu, Xiaoping Ouyang, Jie Wang, Jiangyu Li, Peng Gao

**Affiliations:** 1 International Center for Quantum Materials, Peking University, Beijing, China; 2 Electron Microscopy Laboratory, School of Physics, Peking University, Beijing, China; 3 Department of Engineering Mechanics, School of Aeronautics and Astronautics, Zhejiang University, Hangzhou, China; 4 School of Materials Science and Engineering, Xiangtan University, Xiangtan, Hunan China; 5 Hunan Provincial Key Laboratory of Intelligent Sensors and Advanced Sensor Materials, School of Physics and Electronics, Hunan University of Science and Technology, Xiangtan, Hunan China; 6 Shenzhen Key Laboratory of Nanobiomechanics, Shenzhen Institutes of Advanced Technology, Chinese Academy of Sciences, Shenzhen, Guangdong China; 7 Collaborative Innovation Centre of Quantum Matter, Beijing, China; 8 State Key Laboratory for Artificial Microstructure and Mesoscopic Physics, School of Physics, Peking University, Beijing, China; 9 Beijing National Laboratory for Condensed Matter Physics and Institute of Physics, Chinese Academy of Sciences, Beijing, China; 10 Shenzhen Key Laboratory of Quantum Science and Engineering, Shenzhen, China; 11 Key Laboratory of Soft Machines and Smart Devices of Zhejiang Province, Zhejiang University, Hangzhou, China; 12 Department of Materials Science and Engineering, Southern University of Science and Technology, Shenzhen, Guangdong China; 13 Guangdong Provincial Key Laboratory of Functional Oxide Materials and Devices, Southern University of Science and Technology, Shenzhen, Guangdong China; 14 Interdisciplinary Institute of Light-Element Quantum Materials and Research Center for Light-Element Advanced Materials, Peking University, Beijing, China

**Keywords:** Ferroelectrics and multiferroics, Surfaces, interfaces and thin films

## Abstract

Nontrivial topological structures offer a rich playground in condensed matters and promise alternative device configurations for post-Moore electronics. While recently a number of polar topologies have been discovered in confined ferroelectric PbTiO_3_ within artificially engineered PbTiO_3_/SrTiO_3_ superlattices, little attention was paid to possible topological polar structures in SrTiO_3_. Here we successfully create previously unrealized polar antivortices within the SrTiO_3_ of PbTiO_3_/SrTiO_3_ superlattices, accomplished by carefully engineering their thicknesses guided by phase-field simulation. Field- and thermal-induced Kosterlitz–Thouless-like topological phase transitions have also been demonstrated, and it was discovered that the driving force for antivortex formation is electrostatic instead of elastic. This work completes an important missing link in polar topologies, expands the reaches of topological structures, and offers insight into searching and manipulating polar textures.

## Abstract (web summary)

While the role of PbTiO_3_ in PbTiO_3_/SrTiO_3_ superlattices has been widely studied, little attention is paid to possible polar topologies in SrTiO_3_. Here, the authors create atomic-scale vortex–antivortex pairs in the superlattices, expanding the understanding of topological structures.

## Introduction

Both spins and dipoles prefer alignment and often form uniform patterns that are topologically trivial. Nontrivial topologies such as vortices may arise^1,2^, as schematically shown in the Supplementary Fig. 1a, often resulted from delicate energetic balance in confined structures and leading to exotic properties^3–6^. Such topological structures play important roles in condensed matter physics including fluid dynamics^7,8^, superconductivity^9^, and ferromagnetism^10,11^, and they promise alternative device configurations for post-Moore spintronics and electronics^12,13^. Indeed, magnetic skyrmions are actively pursued for high-density data storage^14^, while polar vortices with exotic negative capacitance^4^ may enable ultralow power consumption in microelectronics. Following extensive investigations on a variety of magnetic textures including vortices^5,11^, domain walls^15^ and skyrmions^16^ in the past decades, studies on polar topologies have taken off in recent years, resulting in discoveries of closure domains^17^, vortices^18^, skyrmions^19^, and meron^20^ in ferroelectric materials.

**Fig. 1:**
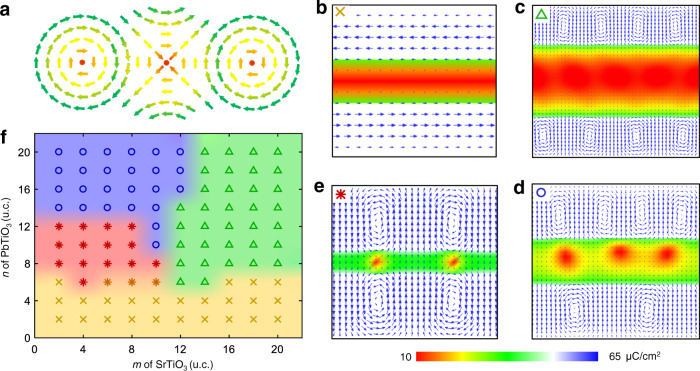
Designing vortex–antivortex pair in (PTO)_*n*_/(STO)_*m*_ superlattice. **a** Schematic illustration of a topological antivortex sandwiched between two adjacent vortices. **b**–**e** Four typical polar structures exist in (PTO)_*n*_/(STO)_*m*_ superlattices for different combinations of *m* and *n*, as predicted by phase-field simulation. **b** For 4-u.c. thick STO (*m* = 4) sandwiched between two 4-u.c. thick PTO (*n* = 4), antiparallel *a*-domain is observed in PTO, while polarization in STO is negligibly small, exhibiting no nontrivial topological structure. **c** For *m* = 20 and *n* = 10, vortex array emerges in PTO, while polarization in STO remains negligibly small. **d** For *m* = 10 and *n* = 10, sign of topological structure appears in STO, with modestly increased polarization, while antivortex appears irregular. **e** For *m* = 4 and *n* = 10, perfect antivortex array with relatively large polarization is observed in STO, sandwiched between two vortices in adjacent PTO. **f** Phase-field computed phase diagram of four typical polar structures in (PTO)_*n*_/(STO)_*m*_ superlattices, as represented by **b**–**e**.

However, creating topologies in polar systems is usually more difficult as the dielectric anisotropy in polar materials is much stronger than magnetic ones^1^, and there is tremendous energy penalty when polarization rotates to form polar topologies. As a result, the atomic-scale polar textures such as closure domains^17,21^, vortices^2,18,22^, and skyrmions^19^ have only been observed in confined PbTiO_3_ (PTO) layers within appropriately designed (PTO)_*n*_/(STO)_*m*_ superlattice (*n* unit cell (u.c.) thick PTO and *m* u.c. thick SrTiO_3_ (STO)). These works mainly focused on PTO within PTO/STO superlattices, which has large polarization, while little attention was paid to the STO. In fact, the cubic structure and paraelectric phase of STO are very delicate to external disturbance^23^. Therefore, it is insightful to examine what happens to the nominally paraelectric STO sandwiched between polar PTO layers. Equally important is if other topological structures often observed in magnetism, such as vortex–antivortex pairs as schematically shown in Supplementary Fig. 1, exist in such dielectric superlattice system or not. Following the groundbreaking work of Kosterlitz and Thouless, it is now well known that vortex–antivortex pair as schematically shown in Fig. 1a may form during Kosterlitz–Thouless transition^24^, which substantially reduce the energy penalty arising from individual vortex and antivortex. Such a vortex–antivortex pair has indeed been observed in superconducting^9^ as well as ferromagnetic systems^25,26^, and it requires a pair of vortices with identical orientation. In (PTO)_*n*_/(STO)_*m*_ superlattice, however, the neighboring vortices in each PTO layer are observed to possess opposite orientations^18,27^, making the topology in between trivial. We thus turn our search for antivortex to STO sandwiched between two layers of PTO instead. This may appear counterintuitive at the first sight, though polar order has indeed been observed in STO before at the reduced thickness^23^ or in a confined heterostrucure^28,29^, and the weaker polarization induced in nominally cubic STO may exhibit weaker anisotropy, facilitating the formation of vortex–antivortex pair.

Motivated by such considerations and guided by detailed phase-field simulations, we design a series of (PTO)_*n*_/(STO)_*m*_ heterostructures, and successfully create vortex–antivortex pairs in (PTO)_10_/(STO)_4_ system, where the vortices exist in the PTO layers and the antivortices exist in the STO layers. To our best knowledge, this is not only an observation of atomic-scale vortex–antivortex pair in a dielectric system, but also a realization of nontrivial polar topology in STO when embedded in a ferroelectric superlattice system. Furthermore, topological phase transition can be induced by either temperature change or electric field, and by examining the energetics of the superlattice, we conclude that the driving force for such antivortex formation is electrostatic, while misfit strain plays a negligible role. The successful creation of previously unrealized atomic-scale vortex–antivortex pair in PTO/STO superlattices expands the reaches of topological structures and completes an important missing link in polar topologies. The work thus sheds considerable insight into the formation of topological polar structures and offers guidance in searching for polar textures.

## Result

### Phase-field simulation

We first seek to create an antivortex as schematically shown in Fig. 1a, sandwiched between two vortices. Such topological structure has been predicted by Mermin from energetic point of view^30^, though its realization has yet to be demonstrated in a dielectric system. We thus consider superlattices with configuration of (PTO)_*n*_/(STO)_*m*_, wherein *m*-u.c. thick STO layer is sandwiched between two *n*-u.c. thick PTO layers. Array of polar vortices has recently been observed in PTO layer of such superlattices^18^, giving us hope that under appropriate design antivortex may emerge in STO sandwiched between two vortices in two adjacent PTO layers. Based on systematic phase-field simulations, we have identified four typical polar configurations (Fig. 1b–e) for (PTO)_*n*_/(STO)_*m*_, enabling us to construct a phase diagram to guide the design (Fig. 1f). When both PTO and STO layers are ultrathin, for example, for *m*, *n* = 4, *a*-domain is observed in two adjacent PTO layers, while STO layer is also slightly polarized (Supplementary Fig. 2), exhibiting no nontrivial topology (Fig. 1b). When they are both relatively thick, for example, *n* = 10 and *m* = 20, nontrivial vortex array emerges in PTO, while polarization in STO remains negligibly small (Fig. 1c). We thus keep *n* = 10 to maintain the desired vortex array in PTO, and reduce the thickness of STO. At *m* = 10, sign of antivortex pattern appears in STO (Fig. 1d), with its polarization magnitude modestly increases, though the topological structure is not regular, and two vortices in adjacent PTO is not well aligned. When thickness of STO is further reduced to 4 u.c., regular antivortex emerges in STO (Fig. 1e), sandwiched between two nicely aligned vortices in PTO, and the magnitude of its polarization increases further as well. This is precisely what we are looking for, fully consistent with theoretical expectation illustrated in Fig. 1a. The window for the vortex–antivortex pair is quite narrow in the phase diagram (Fig. 1f), with thickness of PTO ranging between 8 and 12 u.c. and thickness of STO smaller than 8 u.c. Note that similar superlattices have been studied by Hong et al. ^29^, though their focus was polar configuration of PTO.

### (PTO)_10_/(STO)_*m*_ superlattice design

**Fig. 2:**
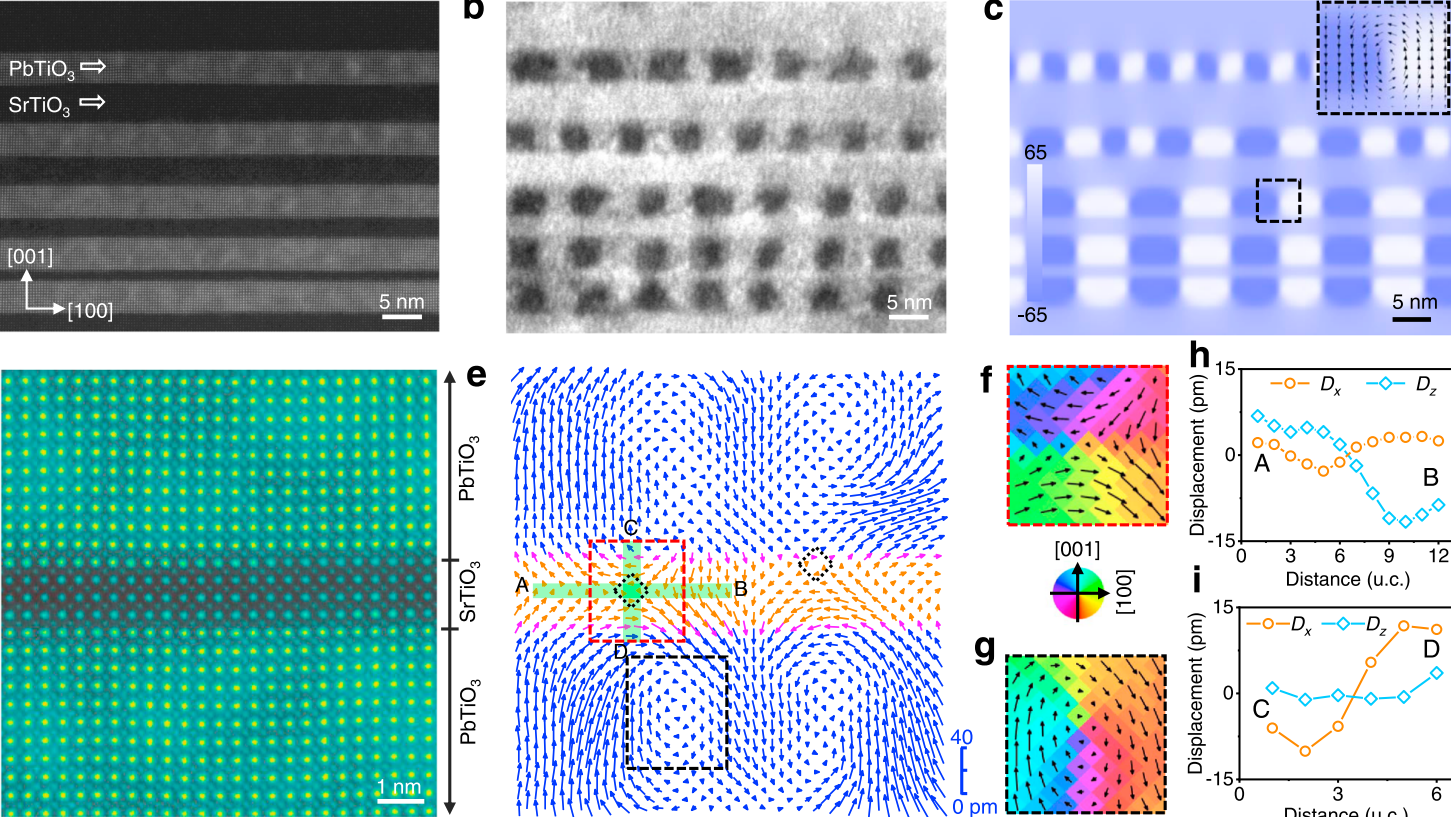
Polar vortex–antivortex pairs in designed (PTO)_*n*_/(STO)_*m*_ superlattice. **a** A low-magnification HAADF image depicts STO layers with varying thicknesses (4, 7, 10, and 15 u.c.) sandwiched between 10-u.c. PTO layers. **b** Dark field TEM image under two-beam conditions by selecting (002)_pc_ **g** vector (subscript pc denotes pseudocubic). The periodic array of bright and dark intensity modulation corresponds to vortex arrays within PTO layers. **c** The spatial distribution of the out-of-plane polarization (unit: μC/cm^2^) was calculated from phase-field simulation. Inset: enlarged view of the polar vector configuration (black arrows). **d** An atomically resolved HAADF image for a 4-u.c. thick STO sandwiched between adjacent 10-u.c. PTO layers, colored for clarity. **e** Map of polar vectors between cations extracted from the HAADF image depicts vortex–antivortex texture. The cores of antivortices are highlighted by the dotted diamond boxes. Enlarged views of polar vectors overlaid with polar angle variation taken from dashed highlighted rectangle boxes in **e** for antivortex (red color) in STO (**f**) and vortex (black color) in PTO (**g**), respectively. Variation of polar displacement components within antivortex structure along A–B (**h**) and C–D (**i**) directions, as marked in **e**. *D*_*x*_ represents in-plane displacement and *D*_*z*_ represents out-of-plane displacement.

Encouraged by phase-field simulation, we design a gradient superlattice heterostructure of (PTO)_10_/(STO)_*m*_, with thickness of PTO fixed at 10 u.c., while that of STO varying at 4, 7, 10, and 15, as shown in Supplementary Fig. 3a. The superlattice heterostructures were then grown on DyScO_3_ (110) substrate by pulsed-laser deposition (PLD). The low-magnification high angle annular dark field (HAADF) scanning transmission electron microscopy (STEM) image of (PTO)_10_/(STO)_*m*_ heterostructure in Fig. 2a illustrates the stacked ferroelectric and dielectric layers of different contrast, whereas Supplementary Fig. 3b–d shows distributions of strain components estimated from geometric phase analysis based on the STEM image. The white-colored sinusoidal wave-like out-of-plane strain pattern is observed within PTO layers along the [100] direction, suggesting the existence of long-range vortex ordering consistent with previous reports^21,22,31^. The dark field transmission electron microscopy (TEM) image shown in Fig. 2b depicts periodic array of bright and dark intensity modulation, corresponding to the clockwise–anticlockwise vortex pairs previously reported in PTO layers^2,18,31^. We can also see such vortex ordering from the spatial distribution of polarization calculated from phase-field simulation (Fig. 2c) that closely resembles Fig. 2b, wherein zoomed-in examination at the interface between dark and bright contrasts clearly reveals a polar vortex.

### Atomic-scale antivortex structure

In order to confirm the polar structure in the superlattice at the atomic scale, we acquired high-magnification HAADF image for 4-u.c. STO sandwiched between 10-u.c. PTO, as shown in Fig. 2d. The *Z*-contrast sensitivity of HAADF imaging shows sharp and coherent interfaces between PTO and STO (*Z* is the atomic number), which is also confirmed by the atomically resolved energy dispersive X-ray spectra mapping incorporated in Supplementary Fig. 4. The polar map (Fig. 2e) of displacement vectors between A site (Pb, Sr) and B site (Ti) derived from HAADF image^32^ illustrates a pair of antivortices within the STO layer, as highlighted by the dotted diamond boxes at their cores, and each antivortex is sandwiched between a pair of vortices in adjacent PTO layers, fully consistent with the theoretical expectation in Fig. 1a. To better appreciate the topology of polar structures, enlarged views of polarization vectors overlaid with polar angle variation for the marked rectangular boxes in STO (Fig. 2f) and PTO (Fig. 2g) are examined, revealing clearly antivortex structure in STO and vortex in PTO. Moreover, the variations of polar displacement within the antivortex along A–B (Fig. 2h) and C–D (Fig. 2i) directions show that out-of-plane (*D*_*z*_) and in-plane (*D*_*x*_) polar vectors reverse their directions when passing through the antivortex core, approaching and departing the core from two sets of opposite directions (head-to-head and tail-to-tail)^26,28,30,33^. Using the experimental data, we also obtain the distribution of winding numbers^34^ (Supplementary Fig. 5a), confirming the topological nature of vortices and antivortices about their respective cores, where two antivortices with winding number −1 between four vortices with winding number 1 are revealed. Additional details on the polar topologies can be found in Supplementary Fig. 5b–d along with phase-field simulations (Supplementary Fig. 5e–g), which show good agreement between experiment and simulation.

**Fig. 3:**
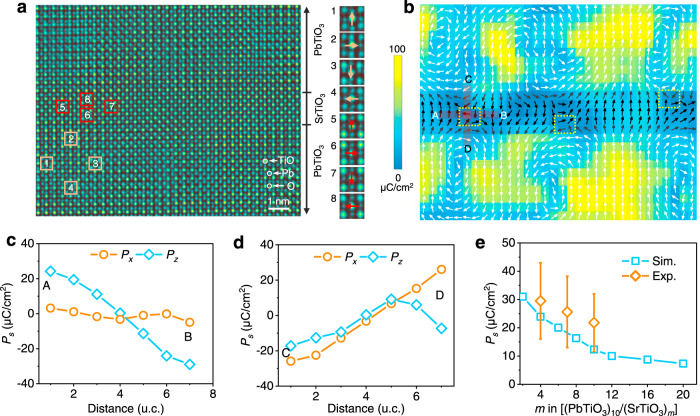
Detailed polarization distribution of polar vortex–antivortex pairs in (PTO)_10_/(STO)_4_. **a** An atomically resolved iDPC image for 4-u.c. thick STO sandwiched between two 10-u.c. PTO layers, colored for clarity. From the enlarged views taken from the marked regions within PTO and STO, the atomic shift between cations and oxygen is visible with the naked eye. **b** The corresponding unit-cell scale map of polarization vectors, calculated from the atomic displacements between cations and oxygen. Arrows denote the polarization orientation and the color represents the magnitude. The yellow dotted boxes highlight the locations of antivortex cores. Variation of polarization along A–B (**c**) and C–D (**d**) directions, as marked in **b**. *P*_*x*_ represents in-plane polarization and *P*_*z*_ represents out-of-plane polarization. **e** The comparison of measured (orange) and phase-field simulated (blue) average polarization versus *m* for (PTO)_10_/(STO)_*m*_. The error bar represents the standard deviation.

The accurate quantitative measurement of polarization in STO, particularly at larger thicknesses, remains a challenge for HAADF image because the polarity in STO mainly arises from the displacement of the oxygen^23^, while HAADF tends to underestimate the STO polarization relative to that of PTO^35^. Thus, we also acquired integrated differential phase contrast (iDPC) image^22^, which presents the information of oxygen configurations with picometer precision and thus gives better accuracy for polarization measurements based on the atomic displacements between cations and oxygen^22^ (see “Methods” for details). From the iDPC image (colored for clarity) in Fig. 3a, the atomic shift between Sr and O for 4-u.c. thick STO with respect to their respective centrosymmetric positions is up to ~20 pm (Supplementary Figs. 6 and 7), visible even with the naked eye. From the enlarged views of atomic structure shown in the inset of Fig. 3a, the octahedron shift in STO (red) are similar to that of PTO (shallow yellow) except less pronounced. The corresponding polar map in Fig. 3b illustrates three antivortices in the STO layer, with their cores highlighted by the dotted boxes. When passing through one of the antivortex cores along either A–B or C–D direction as marked, the polar vectors reverse their directions (Fig. 3c, d) in a similar manner as already revealed by HAADF image (Fig. 2h, i), demonstrating high fidelity of our analysis based on two independent techniques and data sets. The atomic structure of the polarized STO exhibits larger displacement between cations and O and smaller one between Sr and Ti, similar to that of PTO^36^, though its magnitudes of displacements and thus polarization are much smaller (see Supplementary Fig. 7). Indeed, the polarization in the polarized STO is mainly contributed by the oxygen displacement, in good agreement with the previous study^23^. Here, the average magnitude of the polarization is estimated to be ~30 μC/cm^2^ (Supplementary Fig. 7g), consistent with phase-field simulation as well as previous first principle density functional theory calculations^28^. With increased STO thickness, the polarization decreases (Fig. 3e and Supplementary Fig. 8), with the antivortex-like polar topology remaining for 7-u.c. STO, though the structure is less ideal (Supplementary Fig. 9).

### Formation mechanism of polar antivortex

**Fig. 4:**
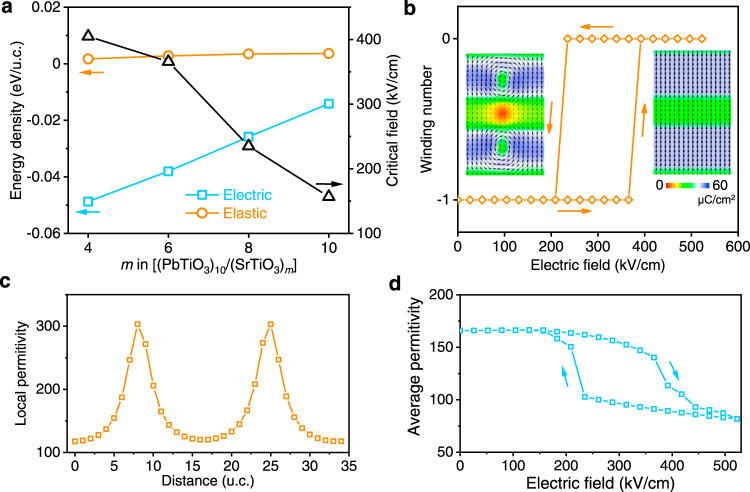
Formation mechanism of antivortex in STO and its electric tuning via phase-field simulation. **a** The electrostatic and elastic energy densities (on the left) for (PTO)_10_/(STO)_*m*_ heterostructures and the critical electric fields (on the right) under which the antivortex disappears. **b** The hysteresis loop of winding number of STO within (PTO)_10_/(STO)_6_ versus external electric field. The left and right insets show the polarization distributions of vortex–antivortex pair and single-domain state, respectively. **c** The spatial distribution of local permittivity in the middle plane of STO across the antivortex cores. The two peaks indicate the significant increase of the permittivity at the antivortex cores. **d** The average permittivity in the middle plane of STO versus the external electric field. The two abrupt changes of permittivity in the hysteresis loop are induced by the topological phase transition.

These two sets of independent STEM data acquired using HAADF and iDPC techniques unambiguously established the existence of antivortex topology in STO, and it is compelling for us to examine its energetics, as shown in Fig. 4a, so that we can understand its formation mechanism. Interestingly, the electrostatic energy density in STO is found from phase-field simulation to be negative and decrease with reduced STO thickness, while the corresponding elastic energy is positive and does not change much with STO thickness. It suggests that the formation of antivortex in STO is largely driven by electric field, while misfit strain in superlattice plays a negligible role. This is in sharp contrast to corresponding analysis for PTO (Supplementary Fig. 10) showing that elastic energy is negative while electric energy is positive, so that the driving force for vortex formation in PTO is elastic, as commonly understood. An immediate implication of this finding is that we may be able to tune the antivortex electrically^37^, as exhibited by the hysteresis loop of winding number versus electric field in Fig. 4b. Reversible field induced topological phase transition is observed, where the vortex–antivortex pair is turned into a single-domain state upon a modest electric field around 392 kV/cm, and is recovered when the electric field is reduced to 209 kV/cm. Similar phase transition can also be induced by heating and cooling (Supplementary Fig. 11), analog to Kosterlitz–Thouless transition^24^. The electric tuning also enables us to examine the stability of antivortex in STO with different thickness, measured by the critical electric field for topological phase transition (Fig. 4a). As expected, this critical field is largest for 4-u.c. STO, demonstrating its highest stability, while that of 10-u.c. STO is substantially reduced and thus is much less stable. Furthermore, the antivortex exhibits positive and much enhanced capacitance at its core, as shown in Fig. 4c, while vortex possesses negative capacitance in excellent agreement with previous report^4^ (Supplementary Fig. 12). Interestingly, the field induced phase transition renders dielectric hysteresis as shown in Fig. 4d, where dielectric tunability as large as 50.7% is observed.

## Discussion

In summary, our work completes an important missing link in polar topology, where stable antivortex is finally confirmed to exist at atomic scale after the discoveries of flux closure, polar vortex, and skyrmions in the artificial PTO/STO superlattices. The small and highly nonuniform polarization may explain why all previous studies did not notice the existence of antivortex in STO. More importantly, it points toward a direction for designing polar topologies in artificial dielectric systems. The energetics of such vortex–antivortex pair is rather delicate, making it nontrivial to realize experimentally, but also make it easier to manipulate via external mechanisms that can easily tip the energetic balances, as we have demonstrated. Our study thus offers a realistic roadmap forward to ultimately engineer and control the polar topologies for devices applications.

## Methods

### Fabrication of designed gradient heterostructures

(PTO)_*n*_/(STO)_*m*_ superlattice heterostructures were grown via PLD (PLD-5000) equipped with a KrF excimer laser (*λ* = 248 nm)^22^. Heterostructures were deposited on (110)-DyScO_3_ substrates by alternately ablating ceramic targets of SrTiO_3_ and Pb_1.1_TiO_3_ at a laser energy of 340 mJ pulse^−1^, and a laser repetition rate of 10 Hz. The substrate was heated to 600 °C in a dynamic oxygen pressure of 200 mtorr for the growth of the PTO and STO layers. Thicknesses of the PTO and STO layers were held at desired thickness through controlling the number of laser pulse. Following growth, the (PTO)_*n*_/(STO)_*m*_ superlattice heterostructures were cooled to room temperature in 200 mtorr oxygen pressure at 10 °C min^−1^.

### TEM cross-sectional sample preparation

For image acquisition, the cross-sectional TEM specimen was thinned to less than 30 μm first by using mechanical polishing. The subsequent argon ion milling was carried out using PIPS^TM^ (Model 691, Gatan Inc.) with the accelerating voltage of 3.5 kV until a hole was made. Low voltage milling was performed with accelerating voltage of 0.3 kV to minimize damage and remove the surface amorphous layer.

### Electron microscopy characterization and image analysis

The dark field TEM image shown in Fig. 2b was carried out under the two-beam condition with **g** vector: **g** = 002_pc_ from an aberration-corrected FEI Titan Themis G2 at 300 kV. HAADF and iDPC images were also recorded at 300 kV using an aberration-corrected FEI Titan Themis G2. The convergence semiangle for imaging is 30 mrad, the collection semiangles snap is 4–21 mrad for the iDPC imaging, and 39–200 mrad for the HAADF. The atom positions were determined by simultaneously fitting with two-dimensional Gaussian peaks using a MATLAB code^32^. The polar vectors in Fig. 2e were plotted from the offset between A site (Pb and Sr) and B site (Ti) sublattices based on the HAADF-STEM image in Fig. 2d. To determine the atomic shift for each atom column in the HAADF, the displacements of A (B) with respect to the center of their surrounding four B (A) columns are measured and decomposed into in-plane and out-of-plane components, respectively. For the iDPC image along [010] direction (Fig. 3a), each cation column [Pb(Sr)] and TiO is surrounded by four oxygen columns. The displacements of cations with respect to the center of their surrounding four oxygen columns can be measured along in-plane and out-of-plane directions, receptively (Supplementary Figs. 6 and 7). Based on the displacements, the unit-scale polarization (Fig. 3b, e and Supplementary Fig. 7g) can be calculated according to *P*_*s*_ = $\frac{1}{V}{\sum} {\delta _iZ_i}$^38^, where *V* is the volume of u.c. which for our case is *a*^2^*c*, *δ* is displacement/shift of atom (*i*) from their centrosymmetric position, and *Z* is the Born effective charge of atom (*i*) calculated by ab initio theory, 6.71 for Ti, 3.92 for Pb, −2.56 for O in PTO, and 7.12 for Ti, 2.54 for Sr, −2.00 for O in STO^38^. Taking the oxygen sublattice as the reference (standard position of each u.c.), the polarization is simplified to be *P*_*s*_ = $\frac{1}{V}( {\delta _{Pb\left( {Sr} \right) - O}Z_{Pb\left( {Sr} \right) - O} + \delta _{Ti - O}Z_{Ti - O}} )$. The vector and magnitude maps of displacement (Fig. 2e) and polarization (Fig. 3b) are plotted by Origin.

### Winding number calculation

We carried out a local winding number analysis for polar angle distribution in order to authenticate the topological nature of vortices and antivortices about their respective cores. The two-dimensional winding number *n* along a closed loop *C* was calculated by the following line integral $n = \frac{1}{{2\pi }}{\oint}_C {\nabla \theta \cdot } \,{\mathrm{d}}r$, where ∇*θ* is the angle gradient of polarization vectors along the integral loop *C*^30,34,39^. By performing a local winding number calculation on each closed loop, we are able to identify the existence of a single vortex and antivortex that gives a winding number equal to +1 and −1, respectively (for illustration simultaneously observe Figs. 2e, f, g and 4b and Supplementary Figs. 5 and 11). We used a condition of |*Δθ*| <180° to determine the angle rotation direction. We have accomplished the winding number quantification for each loop by taking 2 × 2 square loops tilted on the vector map sharing the boundaries.

### Phase-field simulation

In the phase-field modeling, the spatially dependent polarization vector **P** is selected as the order parameter to describe the polar states, and the total free-energy density of a PTO/STO superlattice thin film takes the following form:

1 $$f =	 \, \alpha _iP_i^2 + \alpha _{ij}P_i^2P_j^2 + \alpha _{ijk}P_i^2P_j^2P_k^2 + \frac{1}{2}c_{ijkl}\varepsilon _{ij}\varepsilon _{kl}\\ \, 	- q_{ijkl}\varepsilon _{ij}P_kP_l + \frac{1}{2}g_{ijkl}( {\partial P_i/\partial x_j} )( {\partial P_k/\partial x_l} ) - \frac{1}{2}\varepsilon _0\varepsilon _rE_iE_i - E_iP_i,$$

where *α*_*i*_, *α*_*ij*_, and α_*ijk*_ are the Landau expansion coefficients (sixth- and fourth-order forms for PTO and STO, respectively), *c*_*ijkl*_ is the elastic constant, *q*_*ijkl*_ is the electrostrictive coefficient, *g*_*ijkl*_ is the gradient energy coefficient, *ε*_0_ is the dielectric constant of vacuum, and *ε*_r_ denotes the relative dielectric constant of the background material (cubic PTO and STO in this case). The summation convention for the repeated indices is employed, and the Latin letters *i*, *j*, *k*, and *l* take 1 and 2 in the present work. The detailed expression of each Landau energy forms can be found in the literature^40^. Based on the total free-energy density, the temporal evolution of the polarization field can be obtained by solving the time-dependent Ginzburg–Landau (TDGL) equation

2 $$\frac{{\partial P_i\left( {{\mathbf{r}},t} \right)}}{{\partial t}} = - L\frac{{\delta F}}{{\delta P_i\left( {{\mathbf{r}},t} \right)}}\left( {i = 1,2} \right),$$

where *L* represents the domain wall mobility, $F = {\int}_{V}\; {f{\mathrm{d}}V}$ is the total free-energy, **r** is the spatial position vector, and *t* denotes time. Besides the TDGL equation, both the mechanical equilibrium equation

3 $$\sigma _{ij,j} = \partial \left( {\partial f/\partial \varepsilon _{ij}} \right)/\partial x_j = 0,$$

and the electrostatic equilibrium equation

4 $$D_{i,i} = \partial \left( { - \partial f/\partial E_i} \right)/\partial x_i = 0,$$

must be satisfied simultaneously for a ferroelectric system without body force and space charge.

To solve the above equations, the nonlinear finite element method and backward Euler iteration method are employed for space discretization and time integration, respectively. For clearer illustration and computational simplicity, the carried-out simulations were restricted to the [100]-[001] crystallographic plane, which corresponds to the *x*-*z* plane in the Cartesian coordinate system. Discrete grids with Δ*x* = Δ*z* = 0.4 nm in real space were used for space discretization, and the step length for time integration was chosen as Δ*t*/*t*_0_ = 0.2, where *t*_0_ = 1/(*α*_0_L) and *α*_0_ is the absolute value of *α*_1_ at room temperature. Periodic boundary conditions for the electric potential and polarization components were employed along the *x* direction. The material parameters for PTO and STO used in the simulations are given as follows, based on previous work^40,41^. For PTO, *α*_1_ = 3.8(*T* − 479) × 10^5^ C^−2^ m^2^ N, where *T* is temperature in °C, *α*_11_ = −7.3 × 10^7^ C^−4^ m^6^ N, *α*_12_ = 7.5 × 10^8^ C^−4^ m^6^ N, *α*_111_ = 2.6 × 10^8^ C^−6^ m^10^ N, *α*_112_ = 6.1 × 10^8^ C^−6^ m^10^ N, *α*_123_ = −3.7 × 10 ^9^ C^−6^ m^10^ N, *Q*_11_ = 0.089 C^−2^ m^4^, *Q*_12_ = −0.026 C^−2^ m^4^, *Q*_44_ = 0.03375 C^−2^ m^4^, *C*_11_ = 1.746 × 10^11^ N m^−2^, *C*_12_ = 7.937 × 10^10^ N m^−2^, *C*_44_ = 1.111 × 10^11^ N m^−2^, *ε*_*r*_ = 66, *P*_0_ = 0.757 C m^−2^ is the spontaneous polarization of PTO at room temperature. For STO, *α*_1_ = 7.06(*T*+238) × 10^5^ C^−2^ m^2^ N, *α*_11_ = 1.70 × 10^9^ C^−4^ m^6^ N, *α*_12_ = 1.37 × 10^9^ C^−4^ m^6^ N, *Q*_11_ = 0.0457 C^−2^ m^4^, *Q*_12_ = −0.0135 C^−2^ m^4^, *Q*_44_ = 0.00975 C^−2^ m^4^, *C*_11_ = 3.156 × 10^11^ N m^−2^, *C*_12_ = 1.01 × 10^11^ N m^−2^, *C*_44_ = 1.19 × 10^11^ N m^−2^, *ε*_*r*_ = 100. Note that the electrostrictive coefficients *Q*_ij_ were transformed into *q*_ij_ using the formulas provided by ref. ^41^, and the Landau energy density coefficients were correspondingly modified. In addition, for both PTO and STO, *g*_11_/G_110_ = 0.4, *g*_44_/*G*_110_ = 0.4, where *G*_110_ = 1.73 × 10^−10^ C^−2^ m^4^ N. A normalization process for the material parameters was used to achieve better numerical stability in the simulations, which can be referred to elsewhere^42,43^. The domain structures of suppelattice system are evoluted from initial setup for polarization with small random fluctuation (<0.01*P*_0_). The cubic lattice constants for paraelectric PTO and STO were assumed as 3.955 and 3.905 Å, respectively, hence a misfit strain of −0.3% was applied on the PTO layers in order to take into account the resulted interlayer mechanical inhomogeneity.

The two-dimensional phase-field simulations were carried out for (PTO)_*n*_/(STO)_*m*_ superlattices at room temperature. First, corresponding to the configuration of the gradient (PTO)_10_/(STO)_*m*_ superlattice in the experiment, the phase-field simulations were used to calculate its polarization and strain distributions. In addition, the phase-field calculations were further conducted to complete a phase diagram for the evolution of antivortex states in (PTO)_*n*_/(STO)_*m*_ superlattices. The average polarization (blue color data in Fig. 3e) variation within the STO layer for (PTO)_10_/(STO)_*m*_ superlattice is calculated by $P_{avg} = \left( {\mathop {\sum}\nolimits_{i = 0}^N {P_i} } \right)/N$, where *P*_*i*_ is the polarization magnitude of each node inside STO layer and *N* is the total number of the nodes. For energy density calculation we utilized the formula $f_{avg} = \left( {\mathop {\sum}\nolimits_{i = 0}^M {f_i} } \right)/M$, where *f*_*i*_ *i*s the total energy density of each element inside (PTO)_10_/(STO)_*m*_/(PTO)_10_ superlattice system wherein the subscripts *m* = 4, 6, 8, and 10 u.c as can be seen in Fig. 4a. Furthermore, *f*_i_ is calculated by Gaussian integration based on the Eq. (1) and *M* is total number of elements in the investigated (PTO)_10_/(STO)_*m*_/(PTO)_10_ system. The local permittivity given in Fig. 4c and Supplementary Fig. 12 is calculated by *ε* = Δ*E*_*z*_/Δ*P*_*z*_ after applying a small electric field to the initial stable domain structure without electric field.

## Supplementary information

Supplementary Information

## Data Availability

The data that support the findings of this study are available from the corresponding author upon request.

## Code availability

The MATLAB-based toolbox for fitting atom positions and calculating local polarization is available from the corresponding author upon request.
