# Supplementary material for: Creating polar antivortex in PbTiO3/SrTiO3 superlattice
Source: Nat Commun. 2021 Apr 6;12:2054. doi: 10.1038/s41467-021-22356-0 (PMC8024303; doi:10.1038/s41467-021-22356-0)
Supplement: Supplementary file 1 — Supplementary Information [file 41467_2021_22356_MOESM1_ESM.pdf]

Supplementary Information for:

Creating Polar Antivortex in $PbTiO_3/SrTiO_3$ Superlattice

Adeel Y. Abid[1,2,15], Yuanwei Sun[1,2,15], Xu Hou[3,15], Congbing Tan[4,5,15], Xiangli Zhong[4]*, Ruixue Zhu[1,2], Haoyun Chen[3], Ke Qu[2,6], Yuehui Li[1,2], Mei Wu[1,2], Jingmin Zhang[2], Jinbin Wang[4], Kaihui Liu[7,8], Xuedong Bai[9], Dapeng Yu[7,8,10], Xiaoping Ouyang[4], Jie Wang[3,11]*, Jiangyu Li[6,12,13]* and Peng Gao[1,2,7,14]*

[1]International Center for Quantum Materials, Peking University, Beijing 100871, China.

[2]Electron Microscopy Laboratory, School of Physics, Peking University, Beijing 100871, China.

[3]Department of Engineering Mechanics, School of Aeronautics and Astronautics, Zhejiang University, Hangzhou 310027, China.

[4]School of Materials Science and Engineering, Xiangtan University, Hunan Xiangtan 411105, China.

[5]Hunan Provincial Key Laboratory of Intelligent Sensors and Advanced Sensor Materials, School of Physics and Electronics, Hunan University of Science and Technology, Hunan Xiangtan 411201, China.

[6]Shenzhen Key Laboratory of Nanobiomechanics, Shenzhen Institutes of Advanced Technology, Chinese Academy of Sciences, Guangdong Shenzhen 518055 , China.

[7]Collaborative Innovation Centre of Quantum Matter, Beijing 100871, China.

[8]State Key Laboratory for Artificial Microstructure and Mesoscopic Physics, School of Physics, Peking University, Beijing 100871, China.

[9]Beijing National Laboratory for Condensed Matter Physics and Institute of Physics, Chinese Academy of Sciences, Beijing 100190, China.

[10]Shenzhen Key Laboratory of Quantum Science and Engineering, Shenzhen 518055, China.

[11]Key Laboratory of Soft Machines and Smart Devices of Zhejiang Province, Zhejiang University, Hangzhou, 310027, China.

[12]Department of Materials Science and Engineering, Southern University of Science and Technology, Guangdong Shenzhen 518055, China.

[13]Guangdong Provincial Key Laboratory of Functional Oxide Materials and Devices, Southern University of Science and Technology, Guangdong Shenzhen 518055, China.

[14]Interdisciplinary Institute of Light-Element Quantum Materials and Research Center for Light-Element Advanced Materials, Peking University, Beijing 100871, China.

[15]These authors contributed equally to the work: Adeel Y. Abid, Yuanwei Sun, Xu Hou, Congbing Tan.

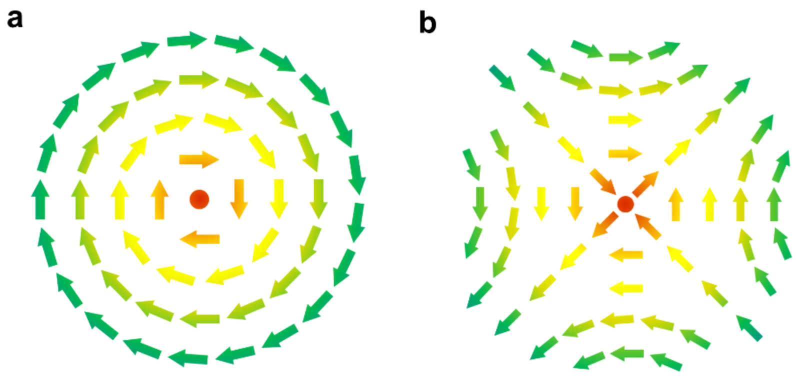

Supplementary Figure 1 | Schematics for a single vortex (a) and antivortex (b).

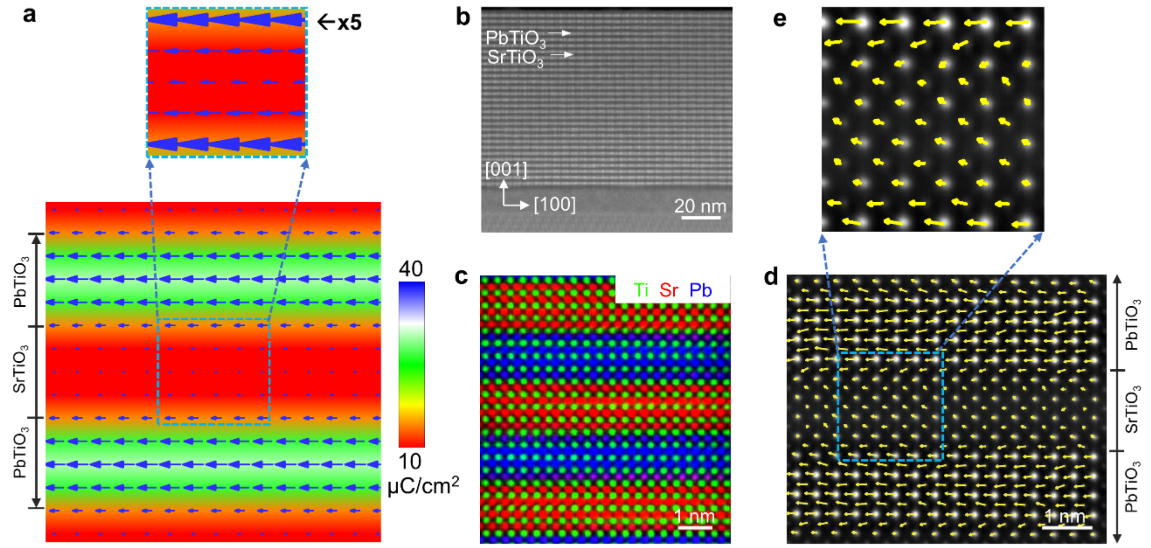

Supplementary Figure 2 | Phase field simulation and STEM images for $(PTO)_4/(STO)_4$ superlattices. **a,** Phase field simulation of $(PTO)_4/(STO)_4/(PTO)_4$ superlattice, the size of arrows inside a selected area of STO layer was magnified 5 times to show the details more clearly. Note that there are two stable configurations for the in-plane polar phase. One is shown in this figure, another is given in Fig.1b. **b,** The low-magnification scanning transmission electron microscopy (STEM) image for $(PTO)_4/(STO)_4$ superlattice. **c,** Atomically resolved elemental mappings of under investigated superlattice with color mix of Ti (green), Sr (red) and Pb (blue). **d,** An atomically resolved HAADF image with overlaid polar vectors of atomic shift between cations. **e,** An enlarged view of a region (for blue squared area in (d)) taken from STO layer with the purpose of showing the polar vector direction more clearly.

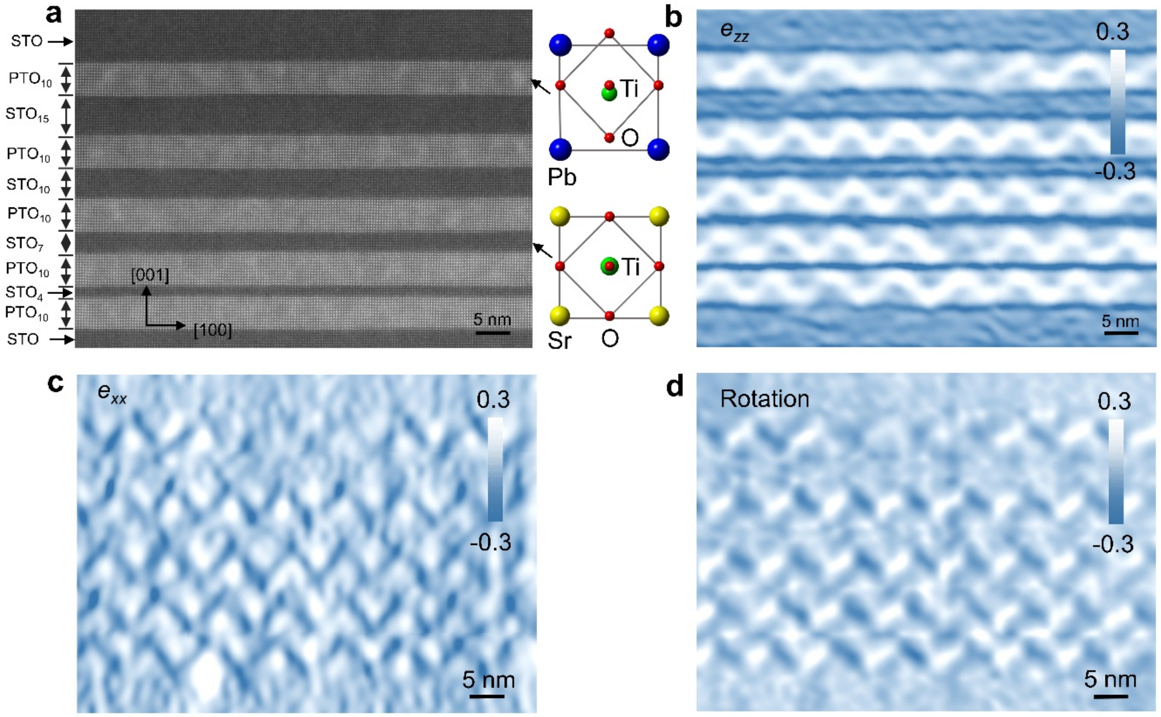

Supplementary Figure 3 | A design of gradient $(PTO)_{10}/(STO)_m$ superlattice. a, The thickness of PTO is fixed at 10-u.c., while that of STO varies at 4, 7, 10, and 15-u.c.. **b,** Out-of-plane strain e_{zz}, **c,** in-plane strain e_{xx}, and **d,** lattice-rotation calculated from the GPA based on the STEM image.

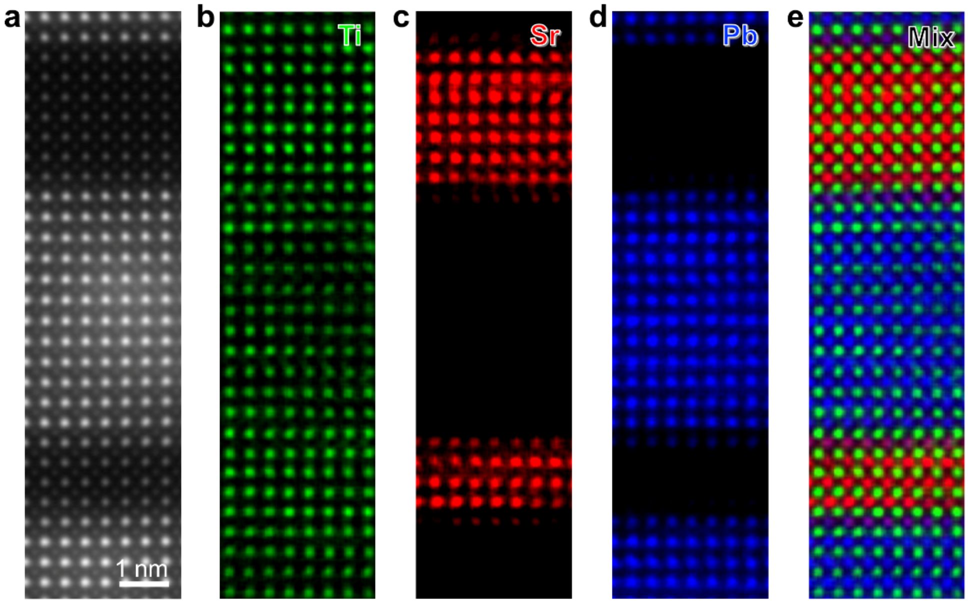

Supplementary Figure 4 | Atomically resolved elemental mappings of the superlattice. a, A HAADF image showing 4 and 7-u.c. thick STO immersed between 10-u.c. thick PTO layers. Net count EDS maps for **b**, Ti (green), **c**, Sr (red), **d**, Pb (blue). **e**, Color mix of Ti, Sr and Pb.

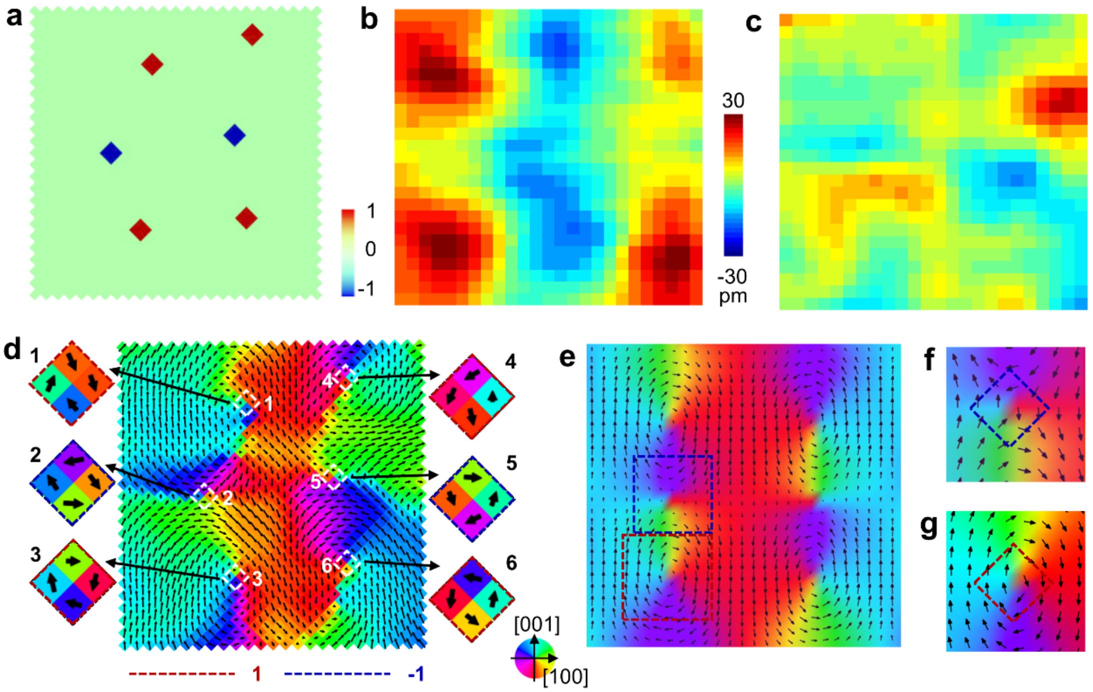

Supplementary Figure 5 | Vortex and antivortex pairs. a, Local winding number calculated from close loop showing winding number +1 and -1 around the core regions of vortices and antivortices (for Fig. 2e) respectively. A two-dimensional color map showing the variation in the magnitude of polar displacements (corresponding to the region in Fig. 2d) for **b,** out-of-plane and **c,** in-plane directions. **d**, A color map of polar angle variation with overlaying polar vectors (black color) for Fig. 2e. The insets highlight the cores of vortex and antivortex. **e,** The corresponding phase field simulated polar angle variation along with enlarged views of antivortex in (**f**) and vortex in (**g**).

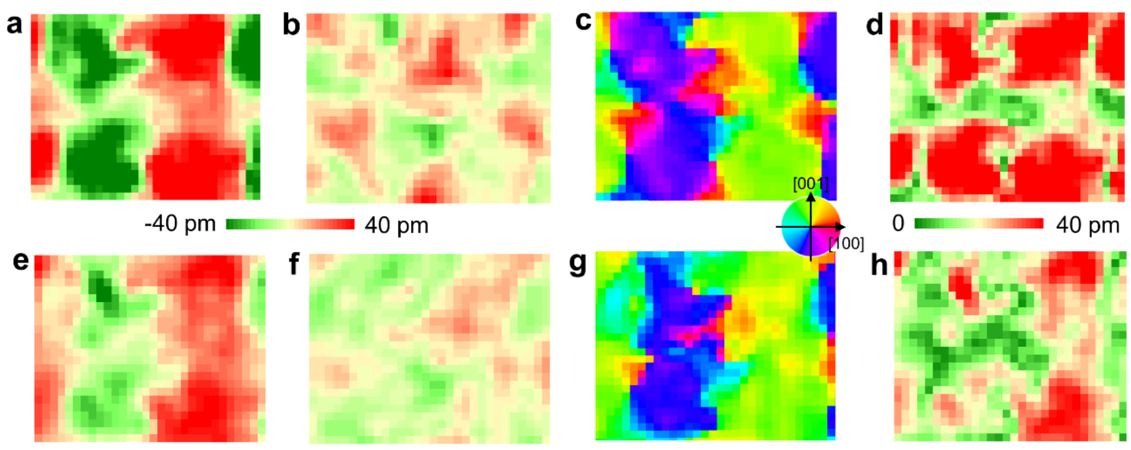

Supplementary Figure 6 | Quantification of displacements between cations and oxygen for $(PTO)_{10}/(STO)_4$. **a**, Out-of-plane, and **b**, in-plane displacements between Pb and O columns. **c**, The orientation, and **d**, magnitude of displacements between Pb and O columns. **e**, Out-of-plane, and **f**, in-plane displacement between TiO and O columns. **g**, The orientation, and **h**, magnitude of displacements between TiO and O columns.

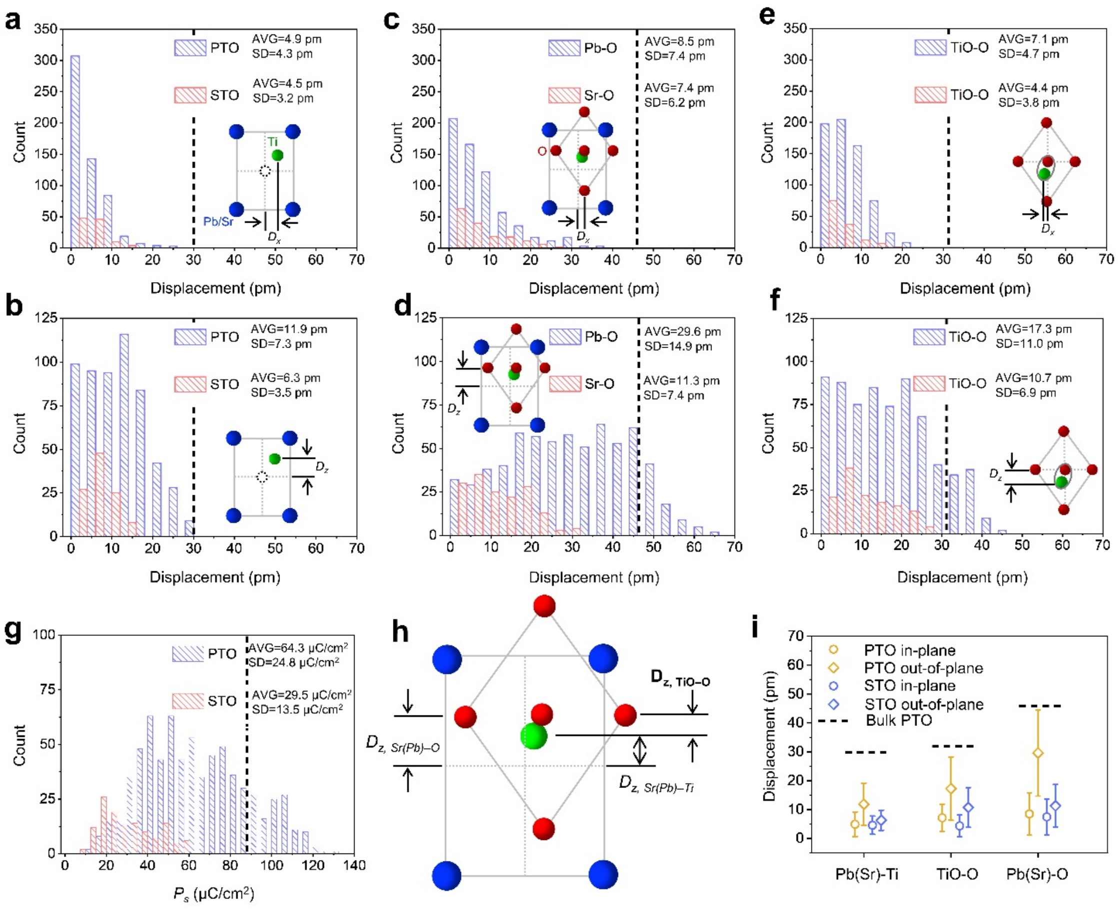

Supplementary Figure 7 | Atomic structure of polarized STO extracted from the displacement measurements. a, In-plane, and **b**, out-of-plane displacements between Pb/Sr and Ti columns extracted from the HAADF image in Fig. 2d. AVG represents for average. SD represents for standard deviation. The vertical black dashed lines denote the values for the bulk PTO from the literature. **c**, In-plane, and **d**, out-of-plane displacements between Pb/Sr and O columns extracted from the iDPC image in Fig. 3a. **e**, In-plane, and **f**, out-of-plane displacements between TiO and O columns extracted from the iDPC image in Fig. 3a. **g,** The distribution of measured polarization of PTO and STO from Fig. 3b. **h,** Schematic showing the atomic structure of polarized STO with the displacements labeled. **i,** The average displacements for STO and PTO in the superlattice $(PTO)_{10}/(STO)_4$. The bulk values of PTO from the literature are also labeled with dashed lines for comparison. The error bar represents the standard deviation.

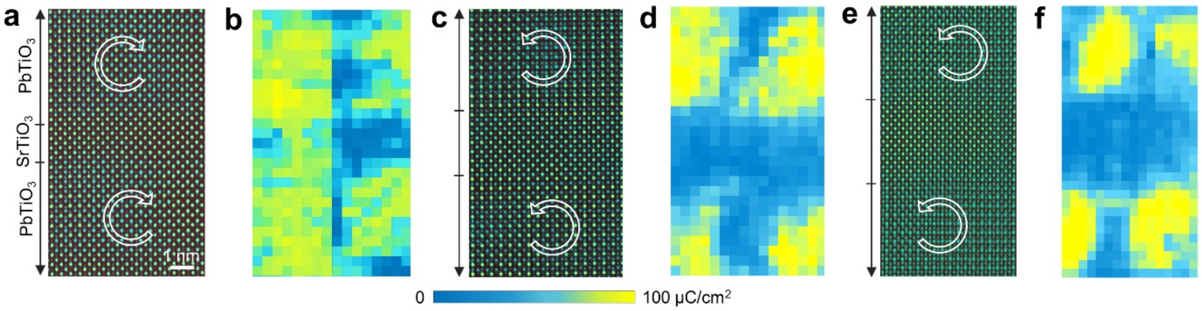

Supplementary Figure 8 | Polarization quantification based on iDPC images for $(PTO)_{10}/(STO)_m$ superlattices (m=4, 7, 10). Atomically resolved iDPC images for **a,** 4, **c,** 7, and **e,** 10-u.c. thick STO layer between 10-u.c. thick PTO layers colored for clarity. The semi-circle arrows indicating the rotating direction of vortices. The corresponding color maps for **b,** 4, **d,** 7 and **f,** 10-u.c. thick STO layer illustrating the polarization variation.

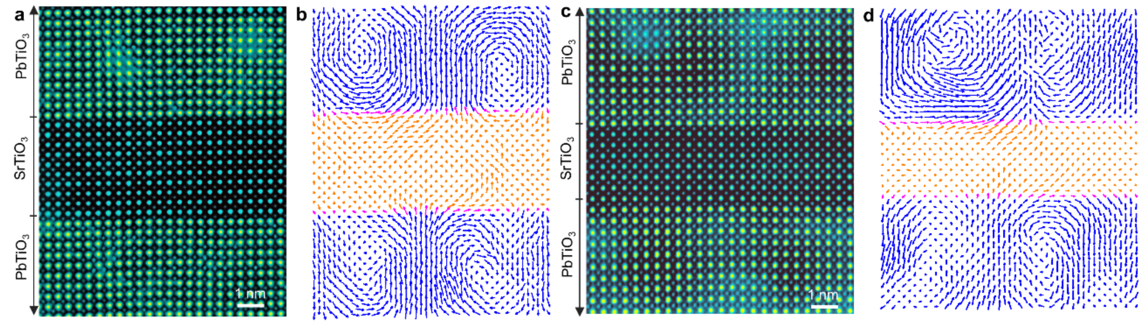

Supplementary Figure 9 | HAADF images with the corresponding polar vectors in $(PTO)_{10}/(STO)_m$ superlattices (m=10, 7). a, An atomically resolved HAADF image for 10-u.c. thick STO layer along with **b**, the corresponding polar vector map. **c,** A HAADF image for 7-u.c. thick STO layer along with **d,** the corresponding polar vector map. Within thick STO layer (7 and 10-u.c.), the polar vectors tend to form an imperfect antivortex structure with a valley-like pattern.

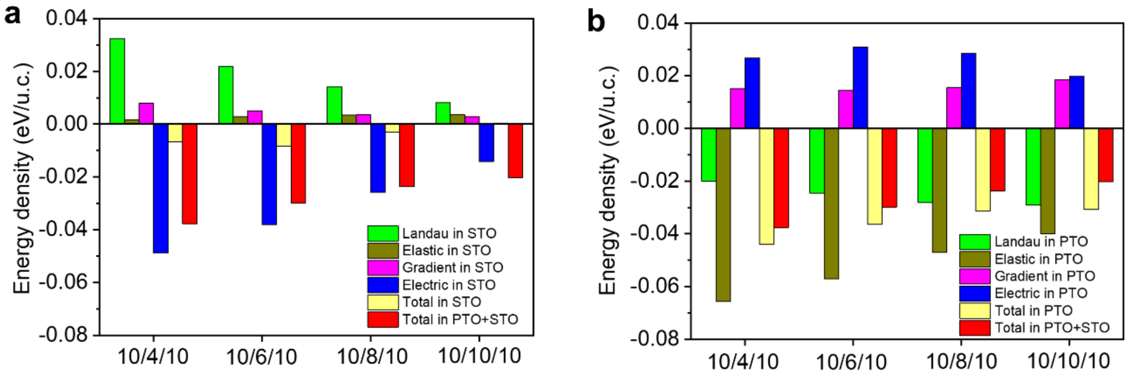

Supplementary Figure 10 | The energy densities in $(PTO)_{10}/(STO)_m$ superlattice. The different energetic terms in the STO (**a**) and PTO (**b**) layers for different m from phase-field simulations.

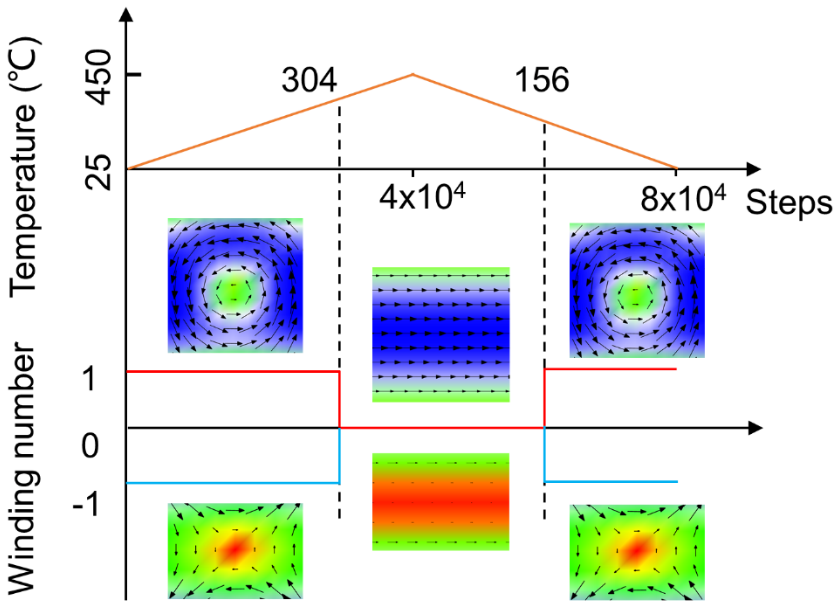

Supplementary Figure 11 | The topological phase transition by temperature changes. The vortex-antivortex pair transfers to a single domain when temperature increases to 304 °C , while the single domain returns to vortex-antivortex pair as temperature decreases to 156 °C. The insets give the polar structures in PTO (above) and STO (below) at different range of temperatures. The corresponding change of winding number in the PTO and STO indicates the topological phase transition.

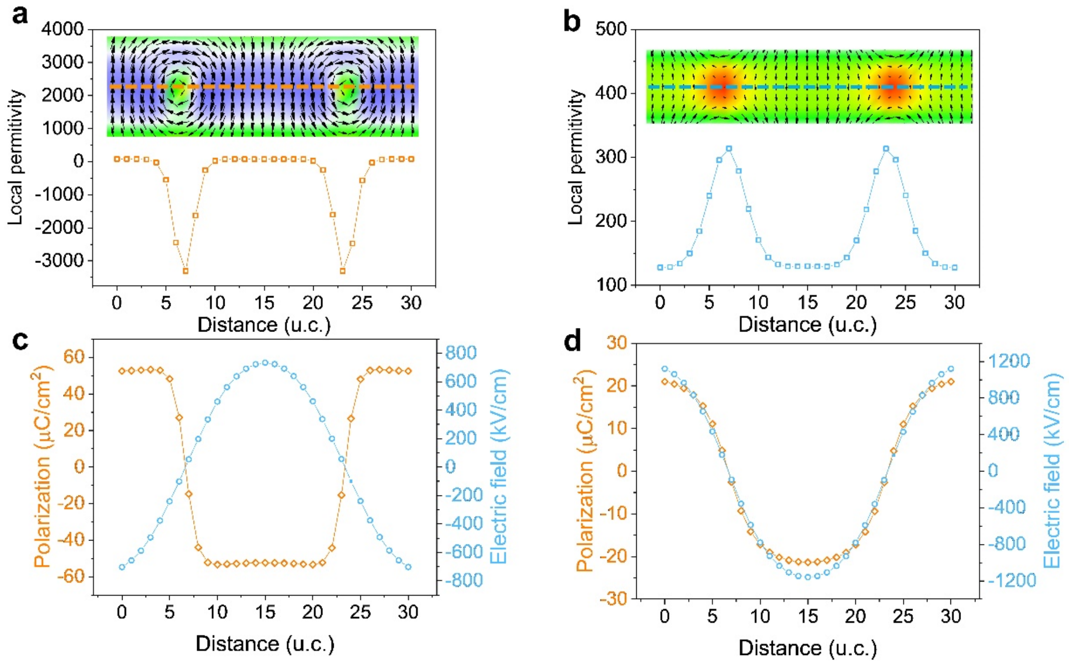

Supplementary Figure 12 | The local permittivity across the vortex and antivortex. The spatial distributions of local permittivity, polarization and electric field in the middle planes of PTO (**a, c**) and STO (**b, d**). Note negative peaks at the vortex cores and positive peaks at the antivortex cores.
